# What do we know about the function of SARS-CoV-2 proteins?

**DOI:** 10.3389/fimmu.2023.1249607

**Published:** 2023-09-18

**Authors:** Santiago Justo Arevalo, Adriana Castillo-Chávez, Carmen Sofia Uribe Calampa, Daniela Zapata Sifuentes, César J. Huallpa, Gianfranco Landa Bianchi, Romina Garavito-Salini Casas, Mauro Quiñones Aguilar, Roberto Pineda Chavarría

**Affiliations:** ^1^ Facultad de Ciencias Biológicas, Universidad Ricardo Palma, Lima, Peru; ^2^ Departmento de Bioquimica, Instituto de Quimica, Universidade de São Paulo, São Paulo, Brazil; ^3^ Facultad de Ciencias, Universidad Nacional Agraria La Molina, Lima, Peru

**Keywords:** immune pathways, COVID-19, pandemic, translation inhibition, viral infection

## Abstract

The COVID-19 pandemic has highlighted the importance in the understanding of the biology of SARS-CoV-2. After more than two years since the first report of COVID-19, it remains crucial to continue studying how SARS-CoV-2 proteins interact with the host metabolism to cause COVID-19. In this review, we summarize the findings regarding the functions of the 16 non-structural, 6 accessory and 4 structural SARS-CoV-2 proteins. We place less emphasis on the spike protein, which has been the subject of several recent reviews. Furthermore, comprehensive reviews about COVID-19 therapeutic have been also published. Therefore, we do not delve into details on these topics; instead we direct the readers to those other reviews. To avoid confusions with what we know about proteins from other coronaviruses, we exclusively report findings that have been experimentally confirmed in SARS-CoV-2. We have identified host mechanisms that appear to be the primary targets of SARS-CoV-2 proteins, including gene expression and immune response pathways such as ribosome translation, JAK/STAT, RIG-1/MDA5 and NF-kβ pathways. Additionally, we emphasize the multiple functions exhibited by SARS-CoV-2 proteins, along with the limited information available for some of these proteins. Our aim with this review is to assist researchers and contribute to the ongoing comprehension of SARS-CoV-2’s pathogenesis.

## Introduction

The outbreak of the COVID-19 pandemic, caused by SARS-CoV-2, has significantly impacted global health, social and economic systems (1–4). SARS-CoV-2 belongs to the family of coronaviruses, which includes other pathogenic viruses such as the severe acute respiratory syndrome coronavirus (SARS-CoV) and the Middle East respiratory syndrome coronavirus (MERS-CoV) (5). SARS-CoV-2 is a single-stranded, positive-sense RNA virus with a genome size of approximately 30 kb, encoding 16 non-structural proteins (nsp1-16), 4 structural proteins (Spike (S), Envelope (E), Membrane (M), Nucleocapsid (N)), and 6 accessory proteins (ORF3a, ORF6, ORF7a, ORF7b, ORF8, and ORF10) (6). While structural proteins form integral components of the SARS-CoV-2 virion, non-structural proteins and accessory proteins facilitate the replication of the SARS-CoV-2 RNA within the cell through different mechanisms.

Here, we present a comprehensive review of the functions described for SARS-CoV-2 proteins. During the first years of the pandemic, significant emphasis was placed on the study of the spike protein of SARS-CoV-2 due to its immunogenicity, surface expression, ability to bind to the human Angiotensin-converting enzyme 2 (ACE2) receptor, and its importance in the vaccine development (7–9). Several reviews have been published with a special focus on topics related to spike protein. Therefore, in this review we just give a brief description of spike functions. For more comprehensive information about this protein, we redirect readers to other reviews (7–9).

Additionally, we would like to note that we have not provided detailed structural information on the extensive array of experimentally and predicted structural models derived for SARS-CoV-2 proteins. For this purpose, we refer readers to an impressive webpage developed by Andrea Thorn’s team which hosts an automatically updated database of SARS-CoV-2 protein structures (10).

Moreover, a significant body of research, employing computational and/or experimental methods, has been conducted to identify potential drugs for treating COVID-19. For an in-depth understanding of these studies, we guide readers to reviews that focus specifically on this topic (11–13). In this review, we mention the proteins that have been considered as potential targets for drug development and provide citations to some of the most relevant literature in this regard.

On the other hand, available reviews on the functions of non-spike proteins are limited, despite their essential role in viral replication, host-cell interactions, and immune evasion. Therefore, this review focuses on the literature describing the functions of SARS-CoV-2 proteins, providing current knowledge on the pathogenesis of SARS-CoV-2. It is important to mention that only confirmed functions in SARS-CoV-2 have been included, and extrapolations from SARS-CoV have not been considered to avoid confusion.

We provide a review of the functions of SARS-CoV-2 proteins and their impact on signaling pathways within the host, including gene expression-translation and the immune system. We expect that this review highlights gaps in our understanding of SARS-CoV-2 pathogenesis and help researches to focus in them.

## Nsp1

Nsp1 consists of two regions: the globular N-terminal domain (1-128 aa) and the C-terminal domain (148-180 aa), which are linked by a flexible region of 20 residues (14, 15). Nsp1 is the produced from the N terminus of the first open-reading frame ORF1a and serves to suppress host gene expression and the immune response (16).

**Figure 1:**
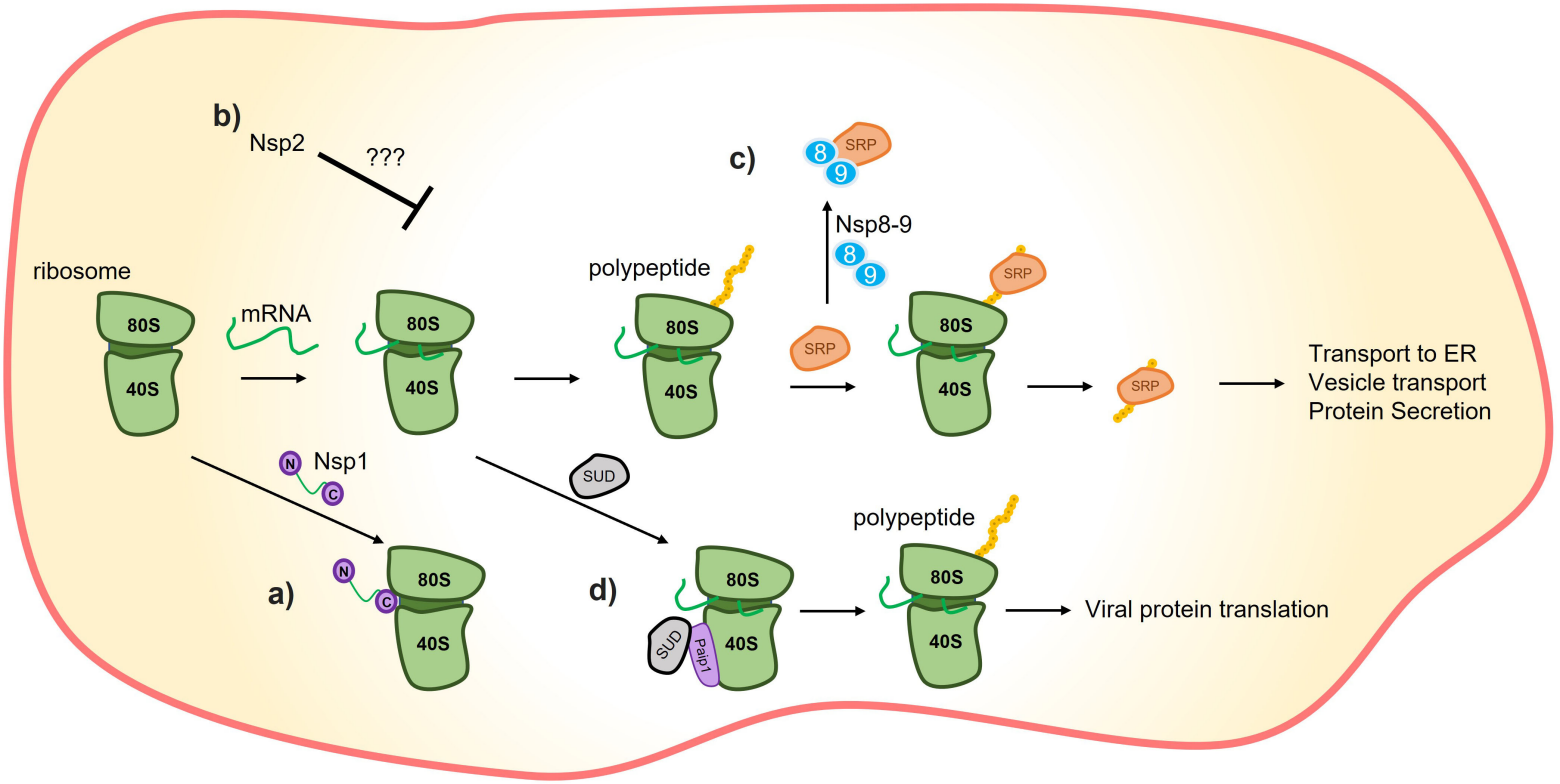
Translation inhibition by SARS-CoV-2 proteins. **(A)** C-terminus of nsp1 induces translation inhibition by binding to the 40S ribosome subunit. **(B)** It has also been demonstrated that nsp2 could promote host-translation inhibition through interactions with translation inhibitors, although the precise mechanism remains unknown. **(C)** Nsp8/9 sequesters the signal recognition particle (SRP), negatively affecting the translation and trafficking of proteins that rely on the SRP mechanism, such as interferons. **(D)** The SARS-unique domain (SUD) of nsp3 can interact with poly(A)-binding protein (PAB)-interacting protein 1 (Paip1) to stimulate translation. The combination of nsp1’s preference for inhibiting host-protein translation inhibition and SUD’s translation-stimulation effect is hypothesized to promote viral translation.

Nsp1 induces a shutdown of host protein translation by binding its C-terminal domain to the 40S ribosome subunit through the conserved residues K164 and H165 (**Figure 1**) (15). This leads to endonucleolytic cleavage and subsequent degradation of host mRNAs (14, 15, 17). This mechanism impacts the immune response by reducing the protein levels, but not the mRNA levels, of Retinoic acid-inducible gene I (RIG-I) and Interferon-stimulated gene 15 (ISG15) (15).

The selective inhibition of host proteins by nsp1 while not affecting viral protein is still under investigation (18). 15, suggest that structural features present in the 5’-UTR of SARS-CoV-2 mRNA may prevent nsp1 from blocking these mRNAs. Supporting this, 19 demonstrated that nsp1 binds to a specific region of SARS-CoV-2 RNA, and 20, showed that stem-loop 1 (SL1) of the 5´ UTR allows SARS-CoV-2 RNA to avoid translation suppression by nsp1. Another possibility is that a higher concentration of viral mRNA outcompetes the translation of the host mRNA, allowing most of the viral mRNA to be translated (21, 22).

## Nsp2

Nsp2 is composed of 638 amino acids and contains three Zinc fingers (ZnFs) in the N-terminal region (PDB: 7EXM, 7MSW) (23). Based on protein sequence alignment of different beta-coronaviruses, the ZnFs in nsp2 are structurally similar to ZnFs of RNA binding proteins. However, despite the presence of a large positive region in nsp2 of SARS-CoV-2 that likely enables interactions with nucleic acids (23), some studies have shown that the ZnFs in SARS-CoV-2 nsp2 are not directly involved in binding to nucleic acids (23, 24).

In general, the function of this protein is not yet well-defined, but it is believed to be involved in viral replication, transcription, and the inhibition of host protein synthesis (**Figure 1**) (23). A conserved interaction (across SARS-CoV-2, SARS-CoV-1, and MERS) has been reported between nsp2 and the proteins GRB10 interacting GYF protein 2 (GIGYF2), Eukaryotic translation initiation factor 4E family member 2 (EIF4E2) and Zinc finger protein 598 (ZNF598), which act as translation inhibitors (24). Additionally, it has been shown by affinity-purification mass spectrometry that nsp2 can interact with other human proteins (**Table 1**) (34); however, further experiments are needed to determine the role of Nsp2’s interactions with these proteins.

**Table 1:** Other proteins that interact with Nsp2 of SARS-CoV-2.

Protein (Abbreviation)	Function	References
Cytochrome p450 oxidoreductase (POR)	Required for electron transfer from NADP to cytochrome P450 in microsomes.	(25)
Solute carrier family 27 member 2 (SLC27A2)	Functions as a long-chain fatty acids transporter and catalyzes the activation of very long-chain fatty acids to their CoA thioesters.	(26, 27)
Rap1 GTPase-GDP dissociation stimulator 1 (RAP1GDS1)	Stimulates GDP/GTP exchange reaction for the Rho family of small GTP-binding proteins.	(28, 29)
Lamin-B2 (LMNB2)	Is thought to provide a framework for the nuclear envelope and may also interact with chromatin.	(30)
Cytochrome b-c1 complex subunit 1 (UQCRC1)	Component of the mitochondrial electron transport chain which drives oxidative phosphorylation.	(31)
WASH complex subunit 4 (WASHC4)	Component of the WASH core complex that functions as a nucleation-promoting factor (NPF) at the surface of endosomes.	(32)
WASH complex subunit 5 (WASHC5)	Component of the WASH core complex that functions as a nucleation-promoting factor (NPF) at the surface of endosomes.	(32)
FK506 binding protein 15 (FKBP15)	Involved in the transport of early endosomes at the level of transition between microfilament-based and microtubule-based movement.	(33)

## Nsp3

Nsp3 is a 1945 amino acid protein that consists of 14 domains: ubiquitin-like 1 domain (Ubl-1), Acidic domain (Ac), X domain (X), SARS-unique domain (SUD), ubiquitin-like 2 domain (Ubl-2), Papain-Like 2 protease domain (PL2pro), Nucleic acid-binding domain (NAB), betacoronavirus-specific marker domain (βSM), transmembrane domain 1 (TM1), nsp3 ectodomain (3Ecto), transmembrane domain 2 (TM2), Amphipathic helix 1 domain (AH1), Y1 domain (Y1), and CoV-Y domain (CoV-Y) (35–37). It is the longest protein encoded in coronaviruses. While the functions of all of its domains are not fully known, different functions have been attributed to this protein. In general, nsp3 is best known for its protease activity that relies in the PL2pro domain, and its essential role in the replication/transcription complex (RTC) (38).

The X domain, also known as Macrodomain 1 (Mac1), removes ADP-ribosylation from the targets of poly(ADP-ribosyl) polymerase 14 (PARP14), a polymerase that promotes antiviral response (39).

Another domain of nsp3 with a described function is the SARS Unique Domain (SUD) which is composed of three subdomains: Macrodomain 2 (Mac2), Macrodomain 3 (Mac3), and Domain preceding Ubl2 and PL2pro (DPUP). These subdomains are also referred to as SARS-unique domain N terminal (SUD_N), middle (SUD_M), and C-terminal (SUD_C), respectively (40). SUD_N-SUD_M (SUD_NM) binds to G-quadruplexes (G4), but the *in vivo* implications of this binding in the pathogenicity of SARS-CoV-2 are yet to be discovered. One possibility is that SUD_NM-G4 interaction regulates the translation of the host cell (36, 40). In this context, studies have demonstrated the interaction between SUD and poly(A)-binding protein (PAB)-interacting protein 1 (Paip1), a facilitator of protein translation (**Figure 1**) (36, 41). This interaction, coupled with the specific host-translation inhibitory effects of nsp1, could enhance viral protein synthesis while repressing host protein synthesis (36).

**Figure 2:**
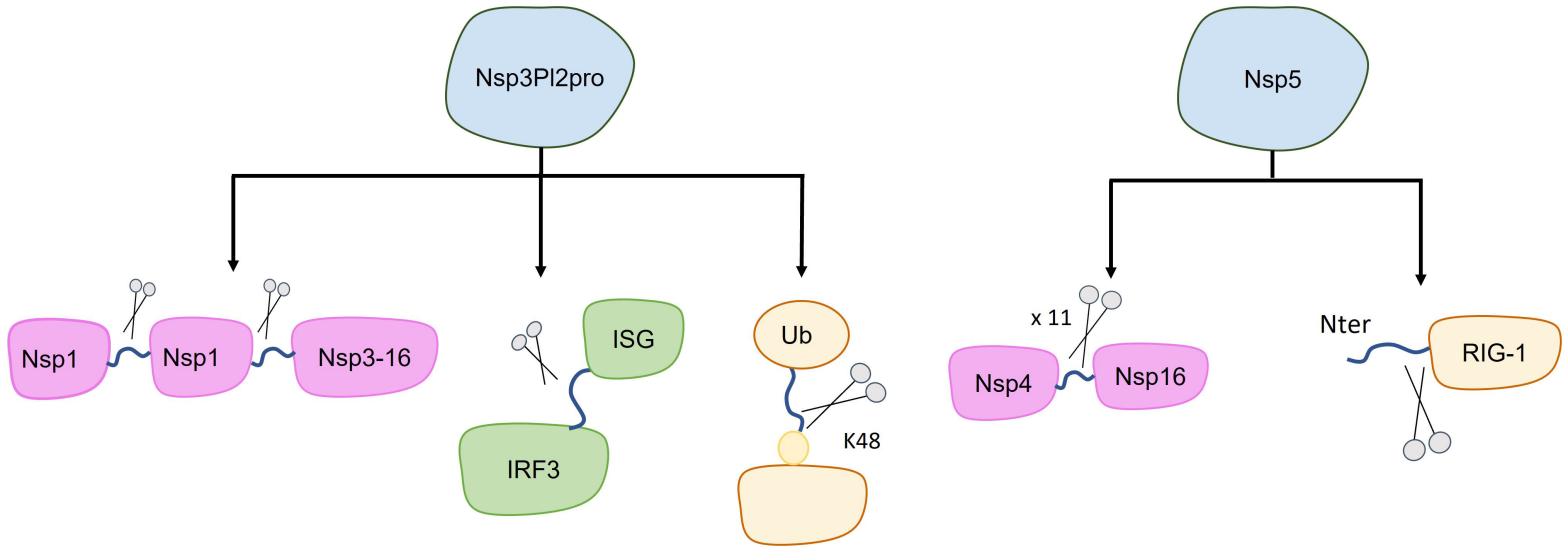
Targets of SARS-CoV-2 proteases. The canonical target of nsp3-PL2pro and nsp5 are the polypeptide 1ab (purple), with each of these proteases cleaving at different sites (indicated by blue lines between purple shapes). Additionally, other cleavage targets have been identified, potentially impacting the inhibition of host immune system (green and yellow). Specifically, nsp3-PL2pro cleaves Interferon Stimulated Gene (ISG-15), leading to the loss of ISGylation from Interferon Responsive Factor 3 (IRF3), and it also cleaves the K48-linked polyubiquitin. Furthermore, nsp5 cleaves the first 10 N-terminal aminoacids from RIG-I, consequently inhibiting MAVS activation.

Due to the homology between PL2pro from SARS-CoV-2 and SARS-CoV, the assigned function to this domain is the processing of the N-terminal of the SARS-CoV-2 polyprotein to release nsp1, nsp2, and nsp3 (**Figure 2**) (42). The cleavage occurs by recognizing the consensus sequence LXGG↓XX. Interestingly, in SARS-CoV-2 it appears that the PL2pro domain alone is not capable of cleaving the viral polypeptide, possibly requiring some other nsp3 component (43).

It has also been shown that PL2pro cleaves Interferon Stimulated Gene 15 (ISG-15), leading to the loss of ISGylation from interferon-responsive factor 3 (IRF3), an important component in the Interferon I pathway (**Figure 2**) (44). If PL2pro of SARS-CoV-2 is able to remove ISG15 from other human targets is yet to be reported. Additionally, PL2pro catalyzes the cleavage of K48-linked polyubiquitin, although with lesser efficiency compared to SARS-CoV (**Figure 2**) (44, 45).

Considerable research has been dedicated to exploring antiviral compounds capable of inhibiting nsp3 functions. The methods employed to investigate these compounds and the identification of the most promising candidates have been thoroughly investigated and reviewed in other sources (11, 46–49).

## Nsp4

Nsp4 is a 500 amino acid protein with 4 transmembrane domains and both termini on the cytoplasmic side (50). It has been shown that nsp4 from SARS-CoV-2 induces changes in the endoplasmic reticulum (ER) structure of the host cell. Specifically, it was shown that Nsp4 and Nsp3 can interact with ER morphogenic proteins reticulon (RTN) likely promoting membrane vesicles curvature (51). Thus, participating in the formation and maintenance of the SARS-CoV-2 replication organelle, which includes double-membrane vesicles and connectors (52). Overall, studies on the function of Nsp4 from SARS-CoV-2, as well as from SARS-CoV, are scarce.

## Nsp5

SARS-CoV-2 nsp5, also known as main protease (Mpro), is a homodimer with three domains: domain I (DI) (8-101), domain II (DII) (102-184), and domain III (DIII) (201-303). Residues 1-7, called N-finger, are located between DII and DIII (53). Nsp5 is a chymotrypsin-related 3C-like protease (3CLpro) that cleaves the polyprotein at 11 sites, generating nsp4 to nsp16. Therefore, inhibition of nsp5 blocks the viral replication cycle (54, 55). Additionally, (56) suggest that the protease activity of nsp5 is essential for viral infection and reorganization of the cellular environment.

**Figure 3:**
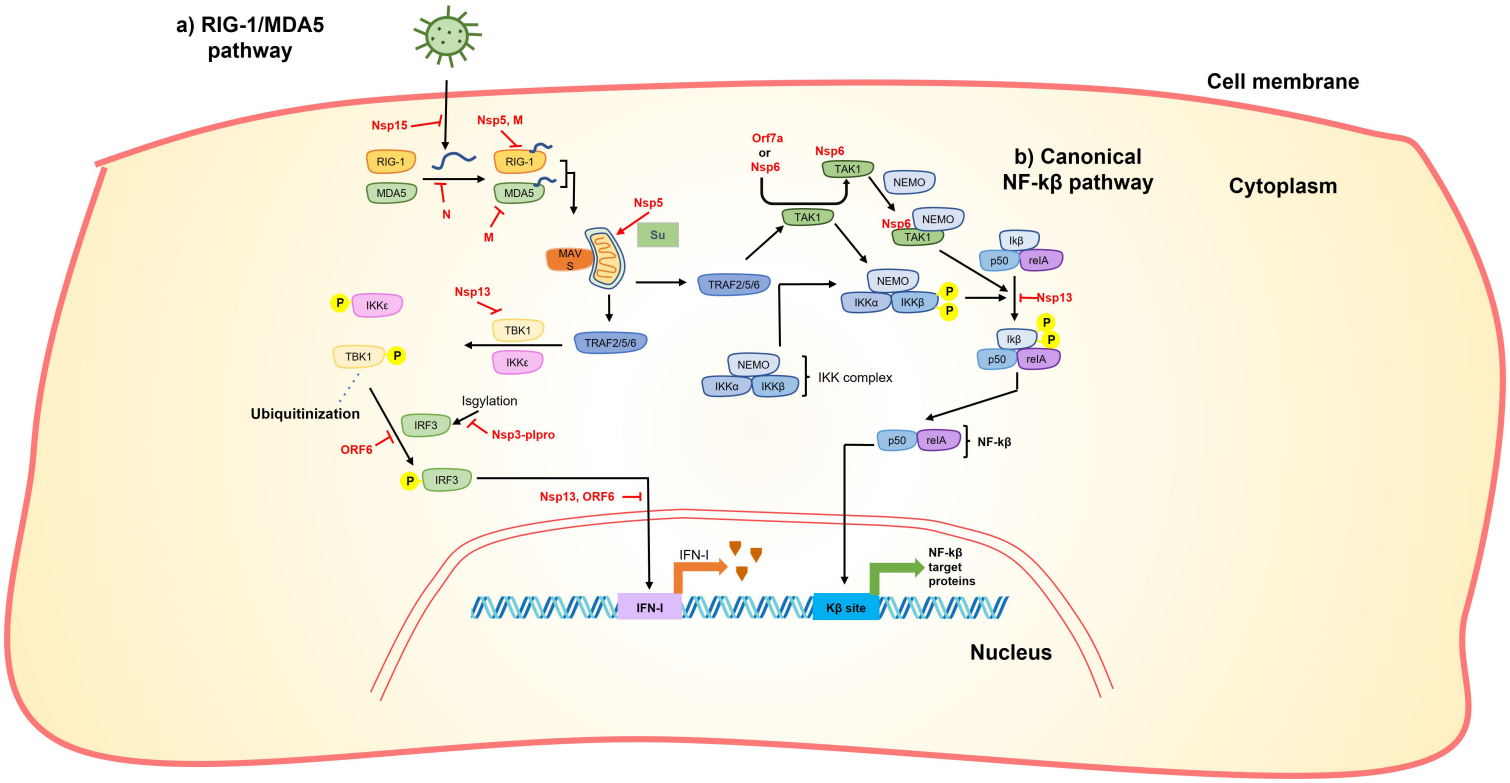
SARS-CoV-2 proteins interfere with RIG-1/MDA5 and NF-kβ pathway. **(A)** Accumulation of viral RNA induces the RIG-1/MDA pathway. This accumulation can be reduced by nsp15 endoribonuclease activity. After its accumulation, RIG-1/MDA5 recognize viral RNA, this step is inhibited by Nsp5, M and N. After viral RNA recognition, RIG-1/MDA5 stabilizes MAVS that subsequently activates TRAF 2/5/6. MAVS can be stabilized by sumoylation catalyzed by Nsp5 promoting expression of inflammatory cytokines. TRAF 2/5/6 phosphorylates IKKϵ and TBK1, this phosphorylation can be inhibited by Nsp13. IKKϵ and TBK1 phosphorylation promotes IRF3 phosphorylation that in turn is translocated to the nucleus to activate IFN-I expression. Phosphorylation of IRF3 is inhibited by ORF6 and IRF3 translocation is interrupted by Nsp13 and ORF6. Additionally, Nsp3plpro cleave ISGylation from IRF3. **(B)** TRAF 2/5/6 also phosphorylates the IKK complex directly or through activation of TAK1. It was shown that Orf7a or Nsp6 interact with TAK1 promoting its interaction with NEMO, this TAK1-NEMO complex promotes phosphorylation of the Ikβ-p50-reIA complex that finally promotes NF-kβ translocation to the nucleus and subsequent activation of NF-kβ target proteins. Thus, nsp6 and orf7a has the ability to stimulate the production of NF-kβ target proteins. On the other hand, nsp13 inhibits the phosphorylation of the Ikβ-p50-reIA reducing the production of NF-kβ target proteins.

Nsp5 has also been shown to act against certain pathways of the host immune response. It has been demonstrated that nsp5 acts as an inhibitor of the retinoic acid-inducible gene 1 (RIG-1)–mitochondrial antiviral signaling (MAVS) protein–interferon (IFN) pathway by proteolytically cleaving the 10 N-terminal amino acids from RIG-I, thereby inhibiting MAVS activation (**Figure 2**) (56, 57). On the other hand, Nsp5 can increase MAVS stability through SUMOylation, activating the Nuclear factor k-light-chain-enhancer of activated B cells (NF-kB) signaling pathway and promoting the expression of inflammatory cytokines (**Figure 3**) (58). Additionally, nsp5 reduce antiviral stress granule (avSG) formation (56).

Similar to nsp3, numerous compounds have been assessed as potent inhibitors of SARS-CoV-2 nsp5. However, delving into details of studies focused on thee inhibitors fall beyond the scope of this review. Therefore, we recommend interested readers to explore these studies elsewhere (11, 54, 59–65).

## Nsp6

Nsp6 from SARS-CoV-2 is a 294 amino acids protein with 6 transmembrane domains (50, 66). Homodimers of nsp6 are localized in the endoplasmic reticulum (ER) forming zippered ER structures that encapsulate the neighboring cytoplasm but selectively allow access to some membrane proteins (66). Thus, nsp6, together with nsp3 and nsp4, connects the double membrane vesicles forming the replication organelle of SARS-CoV-2.

**Figure 4:**
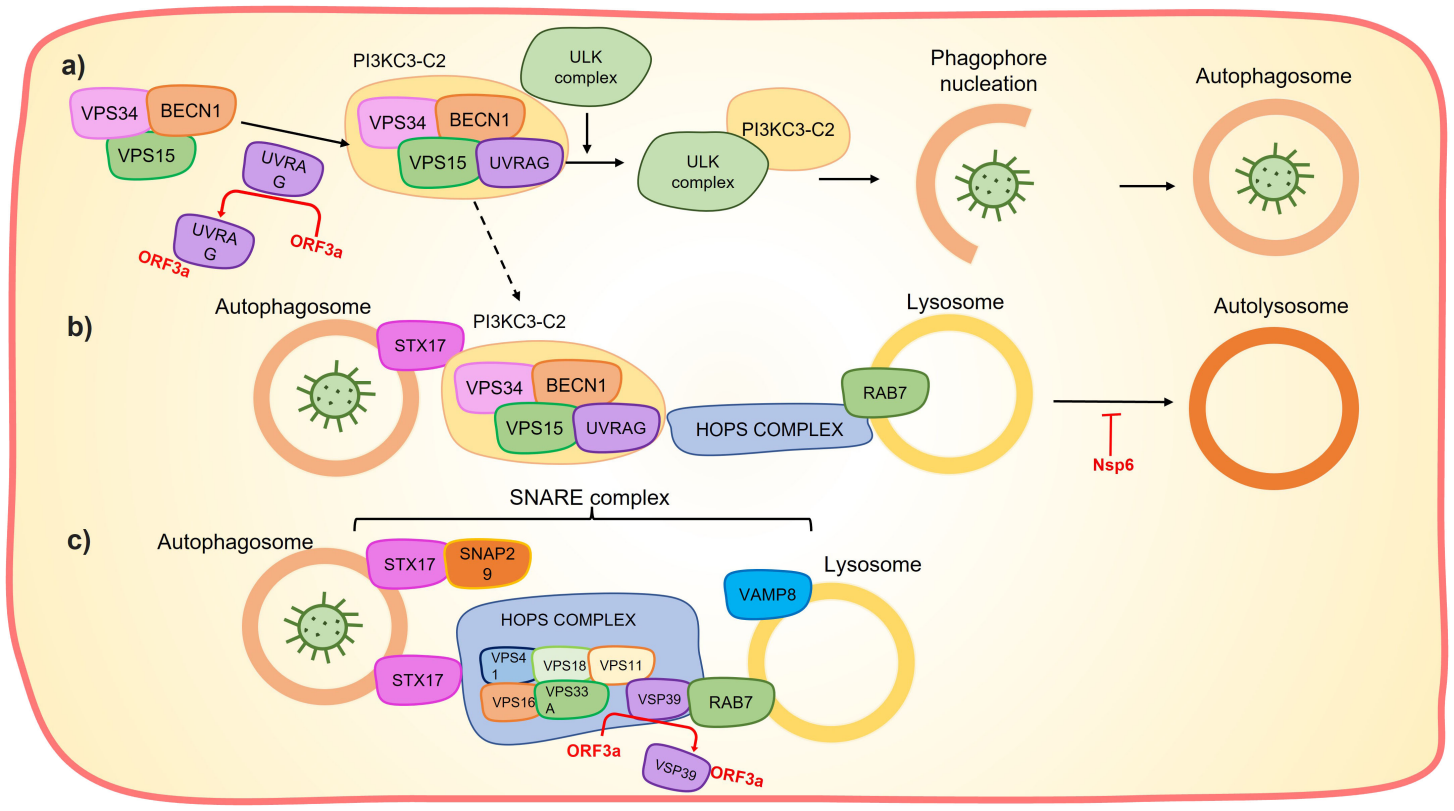
Orf3a and Nps6 affects the autolysosome formation. **(A)** Orf3a sequestrates UVRAG preventing the PI3KC3-C2 formation and the subsequent phagophore nucleation. **(B)** UVRAG sequestration also prevents PI3KC3-C2 interaction with HOPS complex and consequently interferes with the interaction between the autophagosome and the lysosome to form autolysosome. Nsp6 can hinder lysosome acidification which impairs autolysosome formation. **(C)** Orf3a sequestrate the VSP39, which prevents the assembly of the SNARE complex and the subsequent autophagosome-lysosome fusion.

Nsp6 can induce Nuclear factor k-light-chain-enhancer of activated B cells (NF-kB) by recruiting Transforming growth factor beta-activated kinase 1 (TAK1), the TAK1-nsp6 complex then interacts with NF-kβ essential modulator (NEMO), resulting in the activation of NF-kβ signaling pathway (**Figure 3**) (67). The interaction of TAK1-nsp6 with NEMO is dependent on the polyubiquitination of nsp6 at K61, which is carried out by Tripartite motif containing 13 (TRIM13) (67). Furthermore, it has been shown that nsp6 can interact with ATPase H+ transporting accessory protein 1 (ATP6AP1), preventing lysosome acidification and consequently impairing autolysosome formation (**Figure 4**), thereby activating the inflammasome response (68).

Additionally, studies using *Drosophila* cells have shown that Nsp6 can interact with the MAX dimerization protein (MGA/MAX) complex, potentially explaining the COVID-19 associated cardiac pathology (69). However, further studies are needed to confirm this effect in human cell lines.

## Nsp9

SARS-CoV-2 nsp9 is a 113 amino acid RNA-binding homodimeric protein (70). The monomer consists of seven β-strands (β1-β7), an N-terminal β7 extension, and a C-terminal α-helix with a conserved GxxxG motif (PDB ID: 7WQ, 6W9Q) (70, 71). Dimerization of nsp9 occurs through the GxxxG motif, which is also present in SARS-CoV Nsp9 (70–72), as well as the N β7 extension (PDB ID: 7WQ, 6W9Q) (70, 71). It has been suggested that the nsp9 monomer can insert its N terminus into the catalytic center of nsp12 Nidovirus RdRp associated nucleotidyl transferase (NiRAN) domain, forming a bond with GDP that inhibits the nucleotidylation activity of nsp12 (73).

In addition, nsp9 acts as a host virulence factor. Together with nsp8, nsp9 binds to the signal recognition particle (SRP), disrupting its function and suppressing membrane protein trafficking in the host cell. This disruption leads to the suppression of IFN secretion and other cytokines that rely on the SRP complex for secretion (**Figure 1**) (17).

## Nsp10

Nsp10 is composed of 125 amino acids (AA) and consists of two subdomains: the beta subdomain, which includes three antiparallel strands (β1′–β3′), and the alpha helix subdomain, comprising α1–α4 and η1′–η2’, which form two zinc fingers (74, 75). The two zinc-binding sites are as follows: i) C74, C77, C90, and H83 (located between α2 and α3), and ii) C117, C120, C128, and C130. Both sites have stabilizing effects on the protein (75, 76).

Nsp10 forms heterodimers with nsp14 and nsp16 methyltransferases, thereby activating them (76). The interaction interfaces with nsp14 and nsp16 overlap each other, suggesting that the formation of a ternary complex is unlikely (75). The interaction between nsp10 and nsp16 involves hydrophobic interactions and water molecules (76). Two important residues of nsp10, V42 (in pocket 1: M41, V44, V78, A79 and P8O from nsp16) and L45 (in pocket 2: P37, I40, V44, T48, L244 and M247 from Nsp16), are immersed in hydrophobic pockets of nsp16 (76). Furthermore, the interaction between nsp10 and nsp16 apparently induces conformational changes in nsp16, stabilizing more open substrate-binding pockets (77). Notably, pull-down assays have shown that the N-terminal region of nsp10 is crucial for its interaction with nsp14, but not for its interaction with nsp16 (75).

## Nsp 7-8-12

The RNA-dependent RNA-polymerase (RdRp) complex responsible for the replication and transcription of the SARS-CoV-2 RNA is composed of nsp12 (947 aa) and its cofactors, nsp7 (84 aa) and nsp8 (201 aa). In terms of RdRp structure, nsp12 is a 932 amino acid protein that consists of an N-terminal extension domain adopting the nidovirus RdRp-associated nucleotidyltransferase (NiRAN) fold (residues 60 - 49), a polymerase domain (residues 367 - 920), and an interface domain (residues 250 - 365) that connects the polymerase and NiRAN domains (78–80). Additionally, the polymerase domain comprises three subdomains: finger subdomain (residues 366 - 581 and 621 - 679), the palm subdomain (residues 582 - 620 and 680 - 815), and the thumb subdomain (residues 816 - 920) (78–80).

While nsp12 interacts with one turn of a double-stranded RNA, nsp8 has been shown to interact with another turn, located up to 28 base pairs away from the active site. The interaction of nsp8 with the exiting RNA is independent of the RNA sequence and is likely to enhance the processivity of the RdRp complex (79). On the other hand, the exact function of nsp7 is less clear. Konkolova et al. (81) speculated that the tetrameric structure of the nsp7-nsp8 complex could have different functions, such acting as a primase.

In addition to its polymerase activity, nsp12 has been shown to possess nucleotidylation activity in the presence of GTP or UTP, allowing for the formation of the 5´cap structure: GpppA (82). Interestingly, this activity is inhibited when the RdRp is in complex with nsp9 (73).

The RdRp complex has also emerged as a viable target for drug intervention, with at least three drugs having received approval in various countries: Remdesivir, Molnupiravir and JT001 (11). For further information on this topic, we direct readers to several additional publications (83–90).

## Nsp13

Nsp13 is a helicase composed of 603 amino acids that adopts a triangular pyramid shape and presents five domains: Zinc binding domain (ZBD), Stalk (connecting ZBD and 1B), 1B, 1A (RecA-like) and 2A (RecA-like). The nucleotide-binding site is situated in the cleft between the 1A and 2A domains (91–93). Interestingly, the ZBD is capable of binding at least three Zn^2+^ ions through twelve conserved C/H residues (92). The crystal structure reveals three different conformations: two nsp13 molecules in the asymmetric unit (APO form), a phosphate-bound form, and a nucleotide-bound form with AMP-PNP (93). Additionally, there is a pocket linked by domains 1A, 1B, and 2A, which typically binds to the 5′-end of the substrate RNA and is highly conserved, making it a potential pharmacological target (93).

Nsp13 is also involved in the initial step of RNA capping, hydrolyzing the γ-phosphate group of the 5′-terminus of viral RNA. This process prevents recognition by the host immune system (55, 92–94).

**Figure 5:**
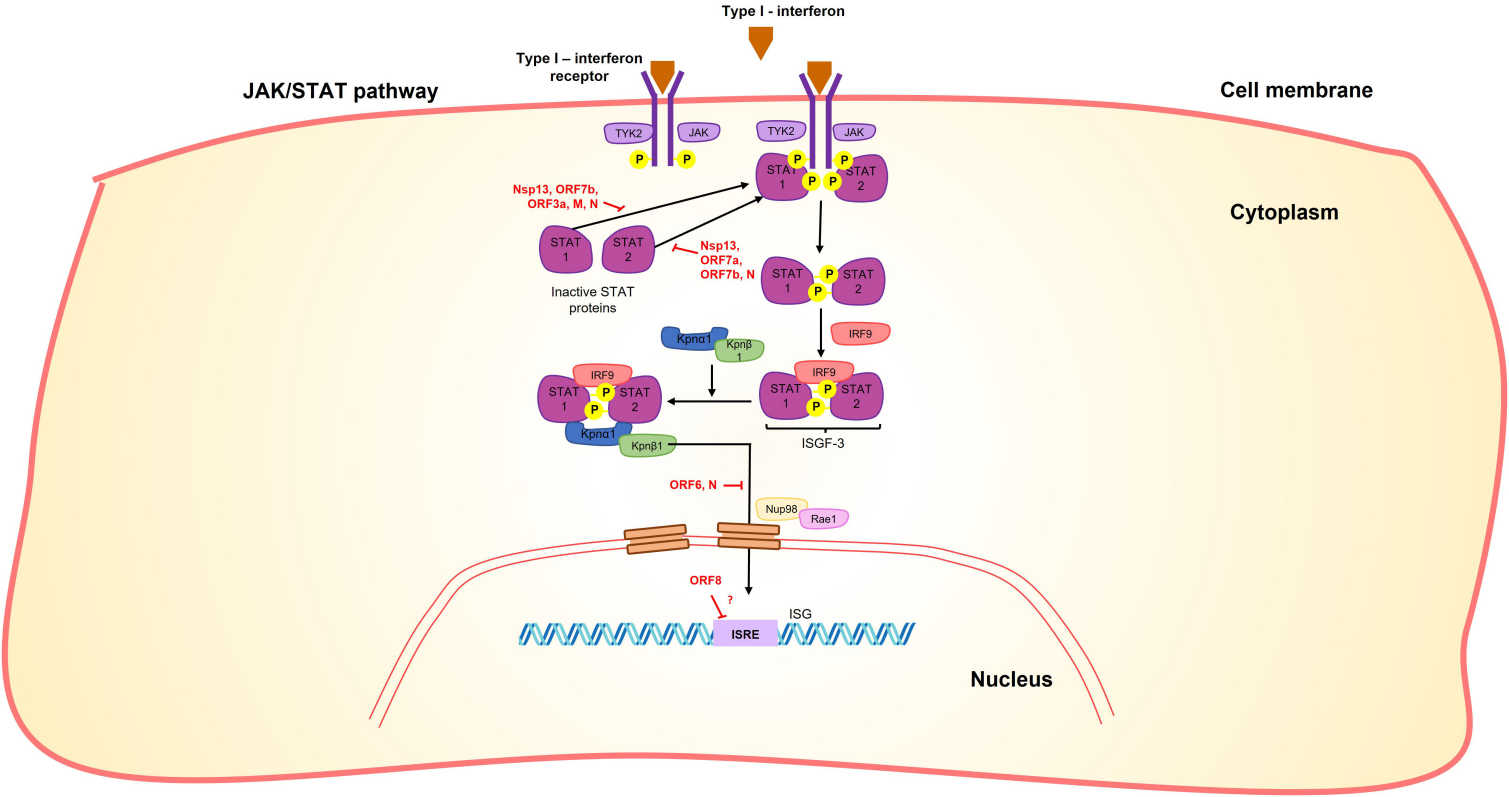
SARS-CoV-2 proteins interfere with the JAK/STAT signaling pathway. Nsp13, Orf7b, Orf3a, M and N proteins interact with STAT1, while Nsp13, Orf7a, Orf7b and N proteins interact with STAT2, in both cases preventing their phosphorylation. N and Orf6 prevent the nuclear translocation of STAT complexes, in the case of ORF6 by interaction with NUP98-RAE1. Moreover, Orf8 blocks the interferon-stimulated response element (ISRE) by an unknown mechanism.

Furthermore, it has been demonstrated that nsp13 inhibits the induction of IFN-I by blocking the retinoic acid-inducible gene 1/melanoma differentiation-associated protein 5 (RIG-1/MDA5) pathway by prevention of TANK-binding kinase 1 (TBK1) phosphorylation (95, 96) and inhibiting nuclear translocation of Interferon regulatory factor 3 (IRF3) (96, 97) (**Figure 3**). It also inhibits the activity of the Interferon-sensitive response element (ISRE) promoter (95, 96, 98) through the suppression of Signal transduce and activator of transcription 1 and 2 (STAT-1 and STAT-2) phosphorylation (95) in the Janus kinase/STAT (JAK/STAT) pathway (**Figure 5**). Moreover, it can inhibit the phosphorylation of NF-kB and its translocation into the nucleus (96) (**Figure 3**).

## Nsp14

The SARS-CoV-2 nsp14 is a bifunctional enzyme conserved among Coronaviridae (99, 100) and is composed of two domains: The N-terminal exonuclease domain (1-287, ExoN) and the C-terminal N7-methyltransferase domain (288-527, N7-MTase) (101).

The ExoN domain functions as a divalent cation-dependent proofreading exoribonuclease, removing mismatched nucleotides from the 3’ end of growing RNAs during RNA synthesis, improving their fidelity (102). On the other hand, the N7-MTase domain, with capping activity, utilizes S-adenosyl-L-methionine (SAM) as the methyl donor to form a cap0 structure (100).

As mentioned above, nsp14 has to be in complex with nsp10 to be active (75). The interaction region of nsp14 with nsp10 involves the ExoN domain but not the N7-MTase domain (75). It has been suggested that the importance of this interaction is to stabilize the ExoN domain in its correct conformation to unleash its proofreading activity (75). Interestingly, the ExoN domain is functional and structurally independent from the MTase domain since the nsp14 ExoN domain bound to nsp10 is enough to show the exonuclease activity (75).

## Nsp15

Nsp15 consist of 347 aminoacids in three domains: The N-terminal domain (NTD), the middle domain (MD), and the C-terminal (CTD) domain (103, 104). Nsp15 forms hexameric structures, where the NTD provides stability, the CTD houses the catalytic site, and the MD serves as a connector between the NTD and CTD (103, 104).

Nsp15 is a nidoviral RNA uridine-specific endoribonuclease (NendoU) that is conserved across all coronaviruses (103, 105). Its primary function is to process viral RNA, specifically cleaving RNA substrates at the 3’ of uridines, thereby evading detection by the innate immune system (104, 105).

Furthermore, nsp15 has been shown to act as an interferon antagonist, inhibiting interferon production following RIG-I activation, although the precise mechanism remains unknown (97). Additionally, it has been suggested that nsp15 suppresses the integral stress response by antagonizing the formation of cytoplasmic stress granules (SGs) through its endoribonuclease activity. This activity reduces the accumulation of viral RNA, thereby preventing the activation of components of the innate immune system such as dsRNA-activated protein kinase R (PKR) or RIG-I-like receptors (RLRs) (**Figure 3**) (106).

## Nsp16

Nsp16 is responsible from methylating the 5’-end of viral mRNAs, generating cap1 structures that mimic cellular mRNAs (74, 107). Similar to nsp14, nsp16 requires nsp10 as an activator (76). Specifically, nsp16 functions as a ribose 2’-O methyltransferase (2′-O-MTase), utilizing S-adenosyl-L-methionine (SAM) as the methyl group donor (76, 107). It has been demonstrated that the activity of nsp16 relies on divalent cations (Mg^+2^ or Mn^+2^) and four conserved residues (D6873, K6874, G6875 and V6876) found across coronaviruses (108).

The active site of nsp16 consists of two main components: i) the catalytic core of the 2′-O-MTase, which adopts a Rossmann-like β-sheet fold and is surrounded by 11 α-helices and 20 loops (74), and ii) the SAM binding site, comprising two crucial regions: a nucleoside pocket and a methionine pocket. The nucleoside pocket forms hydrogen bonds with side chains of D99 and D114, as well as main chains of L100, C115, and Y132. Additionally, water molecules contribute to the hydrogen bonding network with N101 (107). The methionine moiety of SAM forms hydrogen bonds with side chains of N43, Y47, and D130, as well as main chains of G71 and G81 (107).

## Spike

The Spike (S) protein is a trimeric protein expressed on the surface of the virion. It serves as the primary target for recognition by the human receptor ACE2, initiating the viral fusion process with the host cell (109). Due to its surface expression and immunogenicity, it is the primary protein targeted by vaccines (110). Structurally, the spike protein can be divided into two domains: S1 (1-681) and S2 (686-1213) (111). The region between these two domains is known as the S1/S2 cleavage region (682-685) (109, 111). The S1 domain further consists of two subdomains: the N-terminal domain (NTD: 13-303) and the receptor-binding domain (RBD: 319-541) (111). The RBD contains the receptor-binding motif (RBM: 437-508), which directly interacts with the ACE2 receptor (111). The S2 domain includes the fusion peptide (FP: 816-833), heptad repeat 1 (HR: 908-985), central helix (CH: 986-1035), and connector domain (CD: 1076-1141) (111, 112).

Alongside the M protein, the Spike protein is one of the most immunogenic proteins in SARS-CoV-2 (109). Numerous articles have been published during the COVID-19 pandemic focusing on the impact of mutations on the immunogenicity of the spike proteins and its implications for vaccine efficacy. These findings have been summarized in other reviews (7–9).

## Orf3a

ORF3a consists of 275 amino acids and contains a transmembrane domain (TMD) composed of three transmembrane α-helices, which form an ion channel, and a cytosolic domain (CD) with several β-folded sheets (113, 114).

This protein is primarily localized in late endosomes/lysosomes (115) and functions as a potassium ion channel (113). It induces cell death mediated by cellular oxidative stress, including necrosis and apoptosis (116, 117). Orf3a has been associated with reduced cell proliferation (118), decreased cell viability (118, 119) and lysosomal damage (115). Additionally, it promotes viral release through lysosomal exocytosis (120) and is involved in vesicle trafficking (34).

Interestingly, this protein exhibits the ability to inhibit autophagosome formation by blocking phagophore nucleation. It achieves this by sequestering UV radiation resistance associated (UVRAG), leading to the disruption of class III phophatidylinositol 3-kinase complexes I and II (PI3KC3-C2) formation (**Figure 4**) (115, 117, 121–123). The suppression of autolysosome formation through this mechanism may serve to protect the virus. Additionally, autophagosome–lysosome fusion inhibition is accomplished by orf3a through two pathways: i) sequestration of UVRAG, thereby preventing UVRAG-Hepatocyte odd protein shuttling (HOPS) interaction (122); and ii) sequestration of VPS39 (a component of HOPS complex), disrupting the HOPS–Ras-related protein RAB7 interaction and subsequent assembly of the Soluble NSF attachment protein (SNARE) complex (STX17-SNAP29-VAMP8) (115, 117) (**Figure 4**). Interestingly, ORF3a may also promote phagophore nucleation by favoring PI3KC3-C1 formation, likely due to the reduction in PI3KC3-C2 formation (122). Additionally, orf3a has been shown to activate the Unfolded Protein Response, leading to incomplete autophagy via Activating transcription factor 6 (ATF6) and Endoplasmic reticulum to nucleus signaling (IRE1) (123).

Important motifs have been detected in orf3a, including a TNF receptor associated factor 3 (TRAF3)-binding motif (36-PIQAS-40), a caveolin-binding motif (141-YDANYFLCW-149), and diacidic motif (171-SGD-173) (124). Additionally, a cysteine-rich region (81-160) has been observed, which may be involved in the homodimerization of the orf3a. At the C-terminal end, there is a PDZ-binding motif (272-SVPL-275) that has the potential to interact with PDZ-containing proteins (TJP1_2, NHERF4_1, NHERF3_4, RGS3, and PARD3B_1) and potentially impact host cellular functions (124, 125).

Furthermore, orf3a has been shown to promote NF-κB activation and increase the production of Tumor necrosis factor α (TNF-α), Interleukins 1β and 6 (IL-1β and IL-6), and interferon β1 (IFN-β1), potentially through the activation of NF-κB, Toll-like receptor 3 or 4 (TLR3 or TLR4) (114, 118, 126). The deletion of residue G188 leads to an increase in NF-κB activation and downstream cytokines (TNF-α, IL-6, and IFN-β1) (127).

Additionally, orf3a promotes the production of hypoxia inducible factor 1 subunit α (HIF-1α) through the generation of reactive oxygen species in the mitochondria and subsequent mitochondrial damage. This, in turn, enhances the production of pro-inflammatory cytokines (IFN-β, IL-6, and IL-1β) (119). On the other hand, it suppresses IFN- α signaling by inhibiting STAT1 phosphorylation (95) (**Figure 5**).

## Envelope

The Envelope (E) protein is a small viroporin composed of 75 amino acids. It consists of an N-terminal transmembrane domain and a C-terminal domain (128). The transmembrane domain of the E protein has the ability to form homopentameric helices with a vertical length of approximately ~35 Å. Within this helical structure, seven amino acids (N15, L18, L21, V25, L28, A32, and T35) are directed toward the center or the pore, while F23, F26, V29, L31, and I33 stabilize the interfaces between the helices (129). It is worth noting that this homopentameric conformation differs from that observed in the SARS-CoV E protein (130). However, the functional implications of this difference remain unknown.

The C-terminal of the E protein contains a conserved motif (DLLV) that has the potential to interact with Protein associated with LIN7 1 (PALS1), a human cell junction protein. Specifically, this motif could bind to a hydrophobic pocket formed by the PDZ and SH3 domains of PALS1. It is worth noting that the physiological ligand of PALS1, Crumbs C-terminus, interacts with PDZ, SH3 and GK domains (128). PDZ domains play a crucial role in regulating human immune responses (131), suggesting that the interaction between the E protein and PDZ may impact immune system regulation (132, 133).

Furthermore, the E protein functions as a ligand for Toll-like receptor 2 (TLR2), initiating inflammatory signaling pathways such as Extracellular signal-regulated kinases (ERK) and NF-κB, and promoting the production of cytokines including IL-1β, IL-6, and TNF (134). Additionally, the E protein, along with M protein, has an impact on spike protein processing and maturation by promoting its retention in the ER-Golgi intermediate compartment (ERGIC) (135).

## Membrane

The membrane (M) protein of SARS-CoV-2 is composed of 222 residues and is predicted to have three transmembrane domains (136). It is primarly localized in the endoplasmic reticulum (ER) and Golgi (136). Research has demonstrated that the M protein, along with E protein, plays a role in retaining the Spike protein in the ER-Golgi intermediate compartment (ERGIC) through interaction with a C-terminal motif of the Spike protein (135). Additionally, the M and E proteins contribute to the maturation of N-glycosylation of the Spike protein and are necessary for the optimal production of SARS-CoV-2 virus-like particles (135).

The M protein of SARS-CoV-2, along with the Spike protein, is considered one of the most immunogenic proteins (137). The M protein has been shown to inhibit the IFN-antiviral mediated immunity through its interaction with various proteins in the RIG-I/MDA5-MAVS signaling pathway, including RIG-I, MDA5, MAVS, TNF Receptor Associated Factor 3 (TRAF3), Inhibitor of nuclear factor kappa-B kinase subunit epsilon (IKKe), and TANK-binding kinase 1 (TBK1) (**Figure 3**) (136, 138, 139). This interaction leads to the inhibition of phosphorylation and nuclear translocation of IRF3, a transcription factor involved in the production of interferons (136, 138), as well as the stimulation of K48 ubiquitination and subsequent degradation of TBK1, a crucial kinase in the signaling pathway (**Figure 3**) (139).

Furthermore, the M protein negatively affects the phosphorylation of STAT1, further inhibiting the IFN-antiviral immunity (**Figure 5**) (95). Additionally, studies have shown that the M protein can induce apoptosis of lung epithelial cells through its interaction with the BCL-2 ovarian killer (BOK) protein (140).

## Orf6

The orf6 protein of SARS-CoV-2 is predominantly located in the cytoplasm, with partial localization in various cellular compartments such as the Golgi apparatus, endoplasmic reticulum, autophagosomes, and lysosomes (98, 141). Orf6 plays an antagonistic role against the innate immune response by delaying the production and signaling pathway of IFN-β, providing a time window for viral replication (98).

Upon infection, orf6 blocks the activation of interferon regulatory factor 3 (IRF3) through a short peptide sequence in its C-terminal tail (**Figure 3**). This sequence also antagonizes the nuclear translocation of the STAT1 factor (**Figure 5**), which is necessary for the activation of interferon-stimulated response elements (ISRE) (98).

Virus-host protein interaction analyses indicated extensive interactions of orf6 with the nuclear pore complex import/export proteins, such as Nuclear pore protein 98 - mRNA export factor (NUP98-RAE1) or karyopherin subunit α 2 (KPNA2) (**Figure 5**) (34, 141, 142). The interaction of the C-terminal region of orf6 with NUP98-RAE1 and KPNA2 prevents the nuclear import of the STAT and IRF3 complexes (**Figures 3**, **5**) (95, 142). In this way, the ORF6 protein inhibits both the production of IFN-β and its signaling pathway.

## Orf7a

ORF7a is a type I transmembrane protein composed of 121 amino acids. It has a structural organization that includes an N-terminal signal peptide of 15-residues, an 81-residue ectodomain with a compact seven-stranded beta-sandwich similar to members of the immunoglobulin (Ig) superfamily, a 20-residue hydrophobic transmembrane domain (TMD), and a 5-residue ER retention motif (KRKTE) (143, 144).

The ectodomain of orf7a, due to its structural similarity to Ig, is capable of interacting with human CD14+ monocytes, leading to the limitation of antigen presentation (143). Additionally, it induces the production of NF-κB-mediated proinflammatory cytokines such as IL−1α, IL−1β, IL−6, IL−8, IL−10, TNF−α, and IFNβ, as well as other cytokines including IL−3, IL−4, IL−7 and IL−23. Furthermore, orf7a promotes the up-regulation of certain chemokines such as CCL11, CCL17, CCL19, CCL20, CCL21, CCL22, CCL25, CCL26, CCL27 and CXCL9 (126, 143).

Orf7a exhibits other functional properties. It antagonizes the cellular protein Bone marrow stromal antigen 2 (BST-2), which is involved in inhibiting viral egress (145). Additionally, it inhibits the signaling type I interferon (IFN-I) by blocking the phosphorylation of STAT2 (95) (**Figure 5**). This inhibition is facilitated by the ubiquitination of the orf7a (146).

Deletion of specific regions in orf7a, including β5, β6, β7, the transmembrane domain (TMD), and the cytosolic tail, results in the upregulation of various components involved in the IFN-I response. These components include sensors (TLR7), signal transducers (MYD88, OAS2), transcriptional regulators (IRF3, IRF5), and restriction factors (GBP1, IFITM3, MX1). This up-regulation limits the inhibition of the IFN-I response and restricts viral entry (147). Furthermore, this deletion alters the subcellular localization of orf7a from the ER-Golgi intermediate compartment (ERGIC) to the cytoplasm, affecting genomic replication, transcription, and viral egress. However, it does not impact the expression of the contiguous gene, orf7b (147).

## Orf7b

Orf7b is a protein composed of 43 amino acids with a central transmembrane domain. It is capable of localizing in the endoplasmic reticulum (ER), Golgi apparatus, and mitochondria (141, 144).

Orf7b suppress cellular growth, induce cellular hypertrophy, and promote apoptosis via Tumor necrosis factor receptor 1 (TNFR1), TNF-α, and caspase 8 (118, 148). Furthermore, orf7b inhibits signaling of type I interferon by suppressing the phosphorylation of STAT1 and STAT2. It also hinders the nuclear translocation of STAT1 (**Figure 5**) (95).

## Orf8

Orf8 is composed of 121 amino acids and contains an N-terminal signal sequence followed by an Ig-like domain (149). It possesses two distinct dimerization interfaces: i) a covalent interface with a C20 disulfide bridge through a specific N-terminal sequence (115-120) and ii) a non-covalent interface formed by another specific motif (73-YIDI-76) (149).

The N-terminal signal sequence of orf8 can directly interact with major histocompatibility complex (MHC-I) molecules, leading to their downregulation on the cell surface (150). Overexpression of orf8 results in MHC-I downregulation, which serves to protect infected cells from cytotoxic T cells recognition (150). Additionally, orf8 functions as an antagonist of type I interferon (IFN-I) by potentially blocking the interferon-stimulated response element (ISRE) through an as-yet-unknown mechanism (**Figure 5**) (151).

Notably, a mutant form of Orf8 (Δ382) exhibits increased transcript abundance compared to the wild-type, suggesting enhanced evasion of the innate immune system (151). Furthermore, orf8 has been shown to interact with several endoplasmic reticulum proteins, including: EDEM3, ERLEC1, OS9, UGGT2, ERO1B, SIL1, HYOU1, NGLY1, TOR1A and FOXRED2 (34). However, the precise effects of these interactions are not yet well understood.

## Nucleocapside

The nucleocapsid (N) protein is a highly conserved structural protein found in the Coronavirus family (152). It plays crucial roles in viral RNA replication and transcription, as well as in the formation and maintenance of the ribonucleoprotein (RNP) complex.

The N protein is composed of three distinct but highly conserved regions: an N-terminal RNA-binding domain (NTD), a C-terminal dimerization domain (CTD), and a central Ser/Arg (SR)-rich linker that is intrinsically disordered (153, 154). The CTD facilitates the oligomerization of N protein and its interaction with the M protein (155, 156), while the NTD mediates interactions between N protein and viral RNA (153, 154). The SR-rich linker, along with other disordered regions adjacent to the CTD and NTD domains, modulates the oligomerization of the N protein and its interaction with nucleic acids (157).

It has been suggested that the N protein can suppress the IFNβ-mediated immune response by targeting the cellular PRR-RNA-recognition step through the RIG-I pathway (**Figure 3**) (155). Additionally, it acts as an antagonist of type I interferon signaling by inhibiting the phosphorylation and nuclear translocation of STAT1 and STAT2 (**Figure 5**) (158). These functions of the N protein contribute to the immune evasion strategies employed by the virus.

## Orf10

The orf10 protein of SARS-CoV-2 is a relatively uncharacterized protein, and its function and significance are still not well understood. It lacks homology with any known protein, and *in vitro* studies have shown that its deletion does not have a significant impact on viral replication or infection (159, 160). Furthermore, patient cases with mutations in orf10 do not exhibit notable differences in transmissibility or symptoms (159).

Transcriptomic analysis has not detected substantial evidence of orf10 subgenomic reads (21, 161). However, some studies have reported the identification of a transcript that encompasses orf1ab joined to the orf10-3´UTR, suggesting potential roles in RNA stabilization and/or enhancement of nonstructural protein production (162).

*In vitro* experiments have demonstrated an interaction between orf10 and ZYG11B, a substrate of the Cullin-RING E3 ligase complex involved in the ubiquitin-proteasome pathway. This interaction has been shown to impair cilium biogenesis in NIH3T3 and MRC-5 cells by promoting the degradation of an intraflagellar transport complex B protein, IFT46 (163).

## Concluding remarks

The COVID-19 pandemic, caused by SARS-CoV-2, has significantly impacted global health and has become a major challenge for researchers and healthcare professionals. Understanding the functions of the proteins of SARS-CoV-2 is essential for identifying target proteins and/or pathways for the development of new treatments.

As shown, several SARS-CoV-2 proteins, including the structural ones, have more than one function. For example, the proteases nsp3 and nsp5 process the SARS-CoV-2 polypeptide, but they also act against the host immune system by cleaving important signaling molecules. Nsp6 is a structural component of the SARS-CoV-2 replication organelle and, at the same time, it can inhibit the NF-κB signaling pathway by interacting with TAK. Nsp8 is a component of the RdRp that likely increase its processivity. Moreover, it has been shown that nsp8 can modulate the host-translation machinery. Similarly, nsp13 is an RNA helicase involved in RNA replication, but it also affects the host-immune system by inhibiting phosphorylation of TBK1, STAT, and NF-κB. Nsp15 processes the viral RNA but also interferes with the immune response by antagonizing the formation of cytoplasmic stress granules. The structural proteins M and N have also demonstrated effects of interfering with the host immune response. These findings open up the possibility that other SARS-CoV-2 proteins have undiscovered functions.

Throughout this review, it has been shown that several SARS-CoV-2 proteins target host metabolic pathways. Translation is impacted by nsp1, nsp8 and nsp9. Autolysosome formation is targeted by orf3a and nsp6. Immune pathways such as JAK/STAT, RIG-1/MDA5 and NF-κB are affected by multiple SARS-CoV-2 proteins, including nsp3, nsp5, nsp6, nsp13, nsp15, orf3a, M, orf6, orf7a, orf7b, orf8, N. Therefore, it is clear that SARS-CoV-2 has the potential to destabilize overall host metabolism by affecting various mechanisms.

Although the literature on SARS-CoV-2 proteins has grown rapidly, there is still limited information available for several proteins/domains. For example, despite identifying interacting partners for nsp2, its precise function remains unknown. Nsp3 has 14 domains, but only three of them have described functions. Nsp4 and orf10 are other SARS-CoV-2 proteins with poorly understood functions.

By specifically highlighting the confirmed protein functions of SARS-CoV-2, we aim to assist researchers in identifying new targets for further study, thereby contributing to the continual expansion of our knowledge about this pathogen.

## Author contributions

Conceptualization: SJA. Formal analysis: SJA, ACC, CSUC, DZS, CJH, GLB, RGSC, RPC. Visualization: SJA, ACC, CSUC. Writing - original draft preparation: SJA, ACC, CSUC, DZS, CJH, GLB, RGSC, RPC. Writing - review and editing: SJA, ACC, CSUC, DZS, MQA. Supervision: SJA, MQA. All authors read, provided critical review, and approved the final manuscript.

## Publisher’s notes

All claims expressed in this article are solely those of the authors and do not necessarily represent those of their affiliated organizations, or those of the publisher, the editors and the reviewers. Any product that may be evaluated in this article, or claim that may be made by its manufacturer, is not guaranteed or endorsed by the publisher.
